# Inkjet-Printed Graphene-Based 1 × 2 Phased Array Antenna

**DOI:** 10.3390/mi11090863

**Published:** 2020-09-18

**Authors:** Mahmuda Akter Monne, Peter Mack Grubb, Harold Stern, Harish Subbaraman, Ray T. Chen, Maggie Yihong Chen

**Affiliations:** 1 Materials Science Engineering and Commercialization, Texas State University, San Marcos, TX 78666-4684, USA; mam638@txstate.edu; 2 Department of Electrical and Computer Engineering, University of Texas at Austin, Austin, TX 78712-1589, USA; pmgrubb@utexas.edu (P.M.G.); chen@ece.utexas.edu (R.T.C.); 3 Ingram School of Engineering, Texas State University, San Marcos, TX 78666-4684, USA; hstern@txstate.edu; 4 Department of Electrical and Computer Engineering, Boise State University, Boise, ID 83725-0001, USA; harishsubbaraman@boisestate.edu

**Keywords:** graphene, graphene ink, inkjet printing, field effect transistor, true-time delay, phased array antenna

## Abstract

Low-cost and conformal phased array antennas (PAAs) on flexible substrates are of particular interest in many applications. The major deterrents to developing flexible PAA systems are the difficulty in integrating antenna and electronics circuits on the flexible surface, as well as the bendability and oxidation rate of radiating elements and electronics circuits. In this research, graphene ink was developed from graphene flakes and used to inkjet print the radiating element and the active channel of field effect transistors (FETs). Bending and oxidation tests were carried out to validate the application of printed flexible graphene thin films in flexible electronics. An inkjet-printed graphene-based 1 × 2 element phased array antenna was designed and fabricated. Graphene-based field effect transistors were used as switches in the true-time delay line of the phased array antenna. The graphene phased array antenna was 100% inkjet printed on top of a 5 mil flexible Kapton^®^ substrate, at room temperature. Four possible azimuth steering angles were designed for −26.7°, 0°, 13°, and 42.4°. Measured far-field patterns show good agreement with simulation results.

## 1. Introduction

Digital beamforming phased array antennas (PAAs) with distributed control and processing electronics offer numerous advantages for radio frequency (RF) communications, such as electronically controllable beamforming and steering, high average transmitted power and efficiency, flexible sub-arraying to provide multiple communication links simultaneously, reconfigurable high-gain patterns, and increased reliability through reconfiguring redundant array elements. Low-cost and conformal PAAs on flexible substrates are of particular interest in many applications, such as space communications, train antennas, car radio antennas, and cellular base station antennas. 

The major deterrent to producing flexible PAA systems is the difficulty in integrating antenna and electronics circuits on the flexible surface. One of the major integration methods is to attach and interconnect electronic chips onto the antenna surface [1]. Due to the large number of electronic circuits involved in active PAA systems, this attaching and interconnection method is labor-intensive, expensive, unreliable, and impractical for large arrays. Other major approaches of achieving conformal/flexible antennas involve utilizing printed circuit board (PCB)-based conformal antennas [2] and textile-based wearable antennas [3]. Of these, only the last one provides a lightweight, flexible antenna option. However, the need to use external control electronics adds overall weight to the system. Because of this, flexible printed electronics that are capable of large-scale monolithic integration of the electronic circuits with flexible antennas on a single light-weight flexible substrate are highly desired. 

Previously, a prototype PAA was designed, using carbon nanotubes (CNTs) as the active material in the digital switching device and nanosilver ink as interconnect and antenna element material [4,5]. This work not only proved the feasibility of developing a conformal antenna system, but also demonstrated the integration of control electronics on the same substrate at a very low cost. However, there are two major issues concerning the abovementioned technology. First, the silver is easily corroded under harsh environments and long-term use. Second, the CNTs tend to bundle together, limiting the concentration of the ink to 10% by weight and causing the nozzle to clog easily, thus limiting the device performance.

Graphene is a 2D material for electronics and photonics, with remarkable electronic, optical, mechanical, and thermal properties. Graphene is being investigated and applied in many fields [6,7,8,9,10,11,12,13,14,15]. Due to graphene’s flexible, strong, lightweight, transparent, and super-high conductive material properties [6,8,16,17,18,19,20], this material can be foreseen in many fields, for purposes such as hydrogen storage devices and batteries, along with flexible antenna applications [10,11,12,13]. Graphene has good conductivity, is more stable in harsh environments, and is resilient to bending, which makes it a good candidate for flexible electronics. This material also allows faster and smaller transistors with less energy consumption and, in some cases, faster heat dissipation than silicon-based devices. It can also be used in the fabrication of transparent conducting films for solar cell applications, chemical sensors, and in liquid crystal devices [14,21]. Chemically modified graphene sheets are used to fabricate biodevices and label-free DNA sensors [15,22]. By using various conducting, semiconducting, and polymer material inks, electronic devices such as transistors, capacitors, radio-frequency identification (RFID) antennas, and batteries have been fabricated on plastic or paper-like substrates. Research is ongoing to develop functionalities on plastic or paper-like substrates with graphene, which also shows great promise for handling terahertz frequency signals [23].

High-performance graphene powder suitable for inkjet printing has been grown through chemical vapor deposition (CVD) [6]. A single patch antenna was printed with graphene and compared with a silver counterpart [7]. Printed graphene-based field effect transistors (FETs) were reported with on–off ratios of 38 and 2.1 [8,9]. 

Compared to conventional printing techniques, such as offset lithography, gravure printing, flexography, and screen printing, inkjet printing is an ideal solution for high-resolution, low-cost, high-speed printed electronics, due to low material consumption, low equipment and setup costs, and the possibility for mass customization. It is also a non-contact printing technique which does not require any template preparation. The wide application of printed electronics includes organic photodiodes, photovoltaic cells, high-frequency micro-electro-mechanical-switches (MEMS), electronic paper displays, foldable thermochromic displays, and high-performance organic thin-film transistors [7,24,25,26,27]. Last but not least, recent advances in the printed and flexible electronics arena open a whole new realm, widening its application by printing sensors for analysis of ionic concentration, as well as for use in diagnostics applications [28].

Traditional rigid microstrip antennas mainly use copper as the radiating material, due to the cost. However, inks made of nanocopper particles are not mature, and special equipment is needed for the sintering process. The majority of current printed flexible antennas is based on nanosilver particle inks. In this paper, a fully printed 1 × 2 graphene-based PAA is presented. Graphene FETs are used as switches on the TTD lines with on/off ratio of 38 and on-current of 32 µA. Patch antenna elements were also printed with graphene ink. The far-field patterns are characterized and agree well with simulation results. The performance of the graphene-based antenna is compared with the silver-based counterpart. The devices were produced in a room-temperature environment, with thermal curing processes used to anneal the inks which can be substituted with a room-temperature photosintering process. Such a low-temperature processing method permits us to use any flexible substrate with regular transparency, as demonstrated in this paper.

## 2. Materials and Methods 

### 2.1. Graphene Ink Formulation 

To prepare the ink, terpineol was added to cyclohexanone in a volumetric ratio of 15:85. Then, ethyl cellulose was added to the mixture and thereafter placed in an ultrasonic bath (Branson model 2510), which provided mild mechanical vibration and a steady temperature environment for the reaction until all the white powder was completely dissolved. This process takes 1–2 h. Next, the graphene nanoflakes were dispersed in the mixture. For uniform and homogenous dispersion, the nanoflakes were added gradually, at intervals, while the mixture was still in an ultrasonic bath. After the addition of the graphene nanoflakes, the mixture was kept under mild vibration, in the ultrasonic bath, for 10–20 h, and, thereafter, the mixture was centrifuged at 5000 RPM for 20 min, to obtain a homogeneous solution (solids concentration of 2.0 weight%, ~1.7 mg/mL graphene nanoflakes). Finally, the homogeneous dispersant was collected, and the residues were recycled. 

After the preparation of the homogeneous solution of graphene, the viscosity of the solution was measured at different shear rates, using a RheoSense m-VROC viscometer (RheoSense, San Ramon, CA, USA) In the conjugated software module (version: 3.1.4)( RheoSense, San Ramon, CA, USA), we entered the sample size in mL and an estimated viscosity for an ink from the literature. Then the software provided an estimated calculation for minimum and maximum flow rate. We set five different flow rates within the displayed range and measurement times, and then we dispersed the sample through the measurement sensor. Table 1 shows the viscosity measured at different shear rates. The true viscosity was estimated at 5.51 cP, at room temperature (25 °C), based on the best slope fit of *R*^2^ = 0.9967, which is above the minimum regression coefficient of 0.98 that we required for reliable viscosity measurements. This 5.51 cP viscosity at room temperature is compatible with the inkjet printer we used for the whole fabrication process which can jet out ink or solution only if the viscosity of the ink/solution is within the range of 2–12 cP.

**Figure 1:**
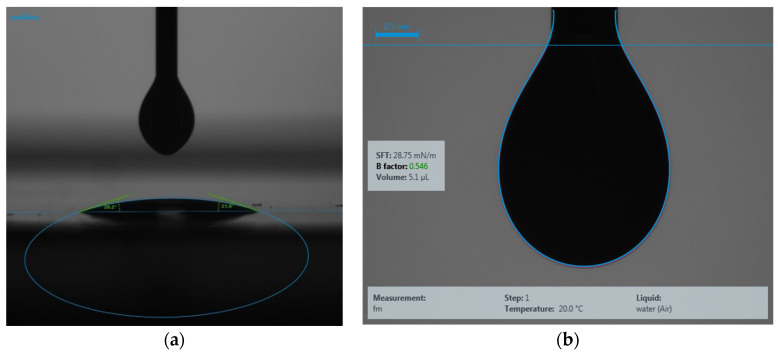
(**a**) Contact angle and (**b**) surface tension measurements between graphene ink with Kapton^®^ substrate.

After the viscosity measurement, the contact angle and surface tension between graphene ink and the substrate were also checked. A KRÜSS drop-shape analyzer (DSA-1000) ( KRÜSS, Hamburg, Germany) was used to determine these two parameters. Contact angle is a crucial characterization to check before any printing technique, including inkjet, aerosol jet, or spray coating. The result of contact angle determines how much resolution we can achieve with an ink, and surface tension determines the bonding between ink and substrate. It is recommended to have a surface tension between 25 and 45 mN/m to achieve good bonding between materials in printing techniques. Figure 1 shows the contact angle and surface tension measurements of the graphene ink with Kapton^®^ flexible substrate. 

The contact angle was found to be between 20 and 21 °C. This contact angle also represents the wettability of the ink with the substrate. The surface tension was found to be 28.75 mN/m, meaning that the ink has a good bonding with flexible Kapton^®^ substrate. 

### 2.2. Inkjet Printing 

A DoD Fujifilm Dimatix (model DMP-2800) inkjet printer (Fujifilm, Santa Clara, CA, USA) was used to print all the thin films and devices. It was equipped with a Dimatix materials cartridge (DMC-11610) (Fujifilm, Santa Clara, CA, USA). The cartridge head has 16 piezoelectric jetting nozzles with a diameter of 21.5 µm each. The nozzles can dispense a droplet of the nominal volume of 10 pL from the cartridge head. The optimum drop spacing for the graphene ink was 30 µm, and six nozzles were used to print these inks. There was no delay between subsequent printing passes, except for a 0.3 s purge in every ten printing passes. Additionally, to avoid the coffee-ring effect, the platen temperature was set at 60 °C [29,30]. The FET device and antenna patterns were created in AutoCAD 2018 (Autodesk, San Rafael, CA, USA). The design patterns were converted into a printable version of the Fujifilm Dimatix printer with ACE 300 software (Numerical Innovations, Las Vegas, NV, USA).

### 2.3. Graphene Film Fabrication 

**Figure 2:**
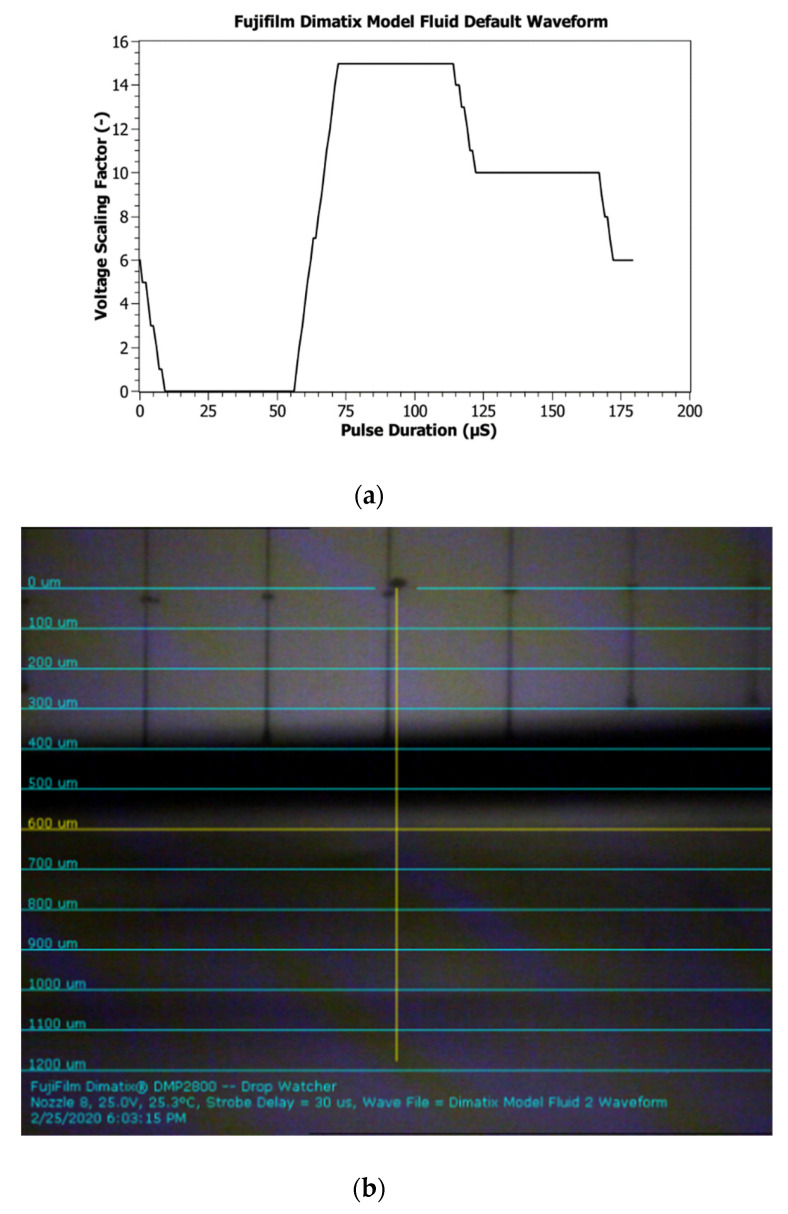
(**a**) Fujifilm Dimatix model fluid default waveform. (**b**) Jetting condition after voltage adjustment.

The selection of an appropriate waveform is important in order to achieve a good printed pattern. Graphene ink is still a new ink concept, and so there are few waveforms available from different vendors, and they do not work perfectly all the time. Thus, the default waveform of Fujifilm Dimatix Model Fluid is a good waveform to start with for any newly formulated ink. Figure 2a,b shows the Fujifilm Dimatix model fluid waveform and jetting condition of graphene ink, respectively. There are three sections in the model fluid waveform: (i) the negative section that draws fluid into the pumping chamber, (ii) the firing pulse with the steepness of the slope providing the energy for the initial ejection, and (iii) the dampening section that prevents the printed head from sucking air back in. This section varies with different material selection.

After investigating the jetting of the nozzles, 5–8 nozzles were chosen to print the antenna. To avoid nozzle clogging, nozzles were cleaned after every 30 min of printing. The final voltage setting for each nozzle was 25 V with 4.5 meniscus setting point. Printing frequency can be selected as low as 1 kHz and as high as 5 kHz. Lower printing frequency increases the printing time, and higher frequency decreases the printing time. However, higher printing frequency is also responsible for significantly increasing the ink spreading all over the substrate. Thus, in our work, the cutoff printing frequency was selected to be 2 kHz, to avoid the ink spreading. The printer head was set at 1.5 mm above the platen, to provide sufficient clearance after placing the substrate. 

Due to the very low percentage (less than 1%) of solid loading of graphene ink, it was necessary to use multiple printing passes. In this work, it was found that, after a minimum of 40 printing passes, graphene could create enough percolation network with good coverage to be conductive. To achieve a good conductive film, thermal annealing was performed after every 10 layers. 

### 2.4. Electrical Characterization 

The current–voltage measurements of the transistors were performed with the Keysight B1500A semiconductor device analyzer (Keysight Technologies, Santa Rosa, CA, USA). In the far-field pattern measurements, the under-test antenna (UTA) was mounted onto a rotational stage, and a standard gain horn antenna was mounted onto a stage with adjustable angles with far-field distance between the two antennas. A vector network analyzer was used to determine S-parameters and provide the RF signal to the UTA. While the UTA was rotated by the rotational stage, a microwave signal analyzer was used to detect the signal from the receiving standard horn antenna, to get the radiation pattern. 

## 3. Results

### 3.1. Graphene Thin-Film Deposition and Reliability Test

Graphene thin film was used to substitute for the widely used silver nanoparticles thin film, due to graphene’s flexibility and anti-oxidization. Graphene nanopowder (Product NP-AO3) (Graphene Supermarket, Ronkonkoma, NY, USA) of 12 nm lateral size was used to prepare printable graphene ink [8]. The printable graphene solution was prepared by using the process described in Section 2. A Fujifilm Dimatix materials printer (DMP-2800) was used to deposit graphene and silver thin films for reliability tests.

#### 3.1.1. Flexibility Test

**Figure 3:**
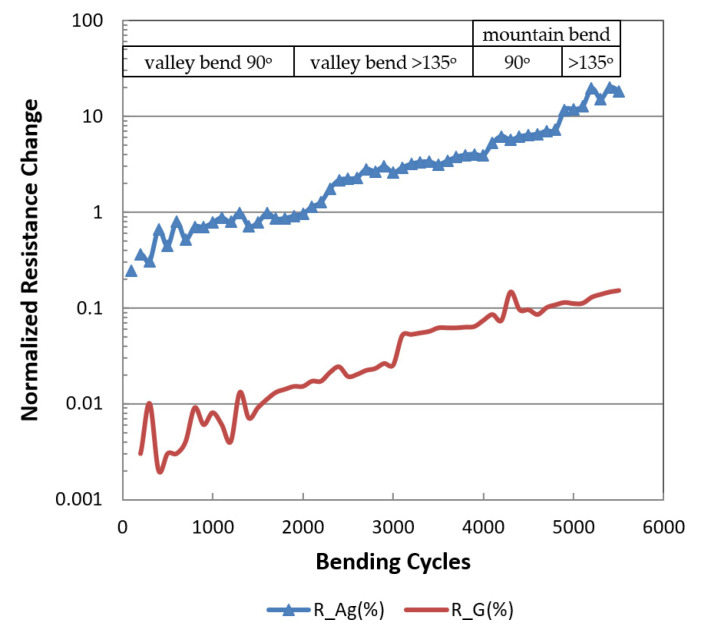
The resistance changes of silver and graphene patches under valley bend of 90° for 2000 bending cycles, followed by 2000 bending cycles of >135° valley bend, 1000 bending cycles of 90° mountain bend, and 500 bending cycles of >135° mountain bend.

The flexibility of the printed graphene and silver patches with the same thickness of one micron were investigated by performing resistance measurements on a homemade bendable system. Valley bend of 90° for 2000 bending cycles was conducted first, followed by 2000 bending cycles of >135° valley bend, 1000 bending cycles of 90° mountain bend, and 500 bending cycles of >135° mountain bend. The resistance of the silver patch increased around 1993% after 5500 bending cycles, while the printed graphene patch increased around 15%. Figure 3 shows the flexible resistance change of inkjet-printed graphene and silver patches.

**Figure 4:**
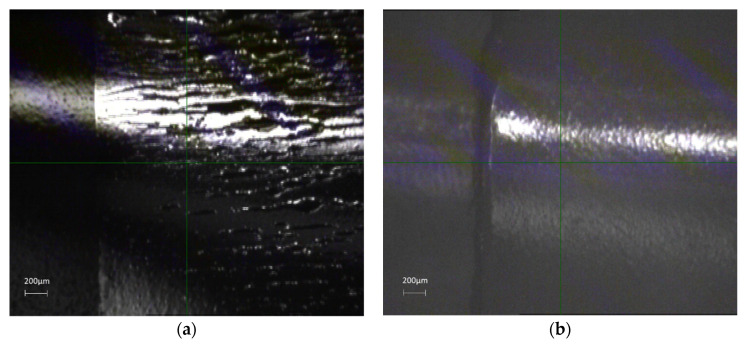
Microscope images of (**a**) silver patch and (**b**) graphene patch after bending test.

Figure 4a,b shows the microscopic images revealing wrinkles on the silver patch and no noticeable wrinkles on the graphene patch. Since the light was shined on the bending line, it does show some structure on the graphene film, due to the bending of the Kapton^®^ substrate. However, the measured resistance data show less of an effect of bending on graphene film. These results indicate that this kind of printed graphene holds a possible pathway for flexible and bendable electronic devices.

#### 3.1.2. Oxidation Test

**Figure 5:**
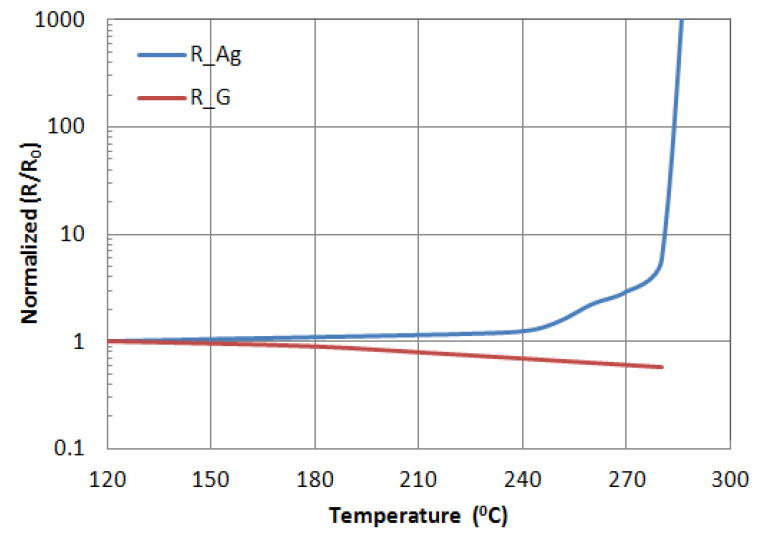
The resistance changes versus heated temperature of silver (R_Ag) and graphene (R_G) patches.

**Figure 6:**
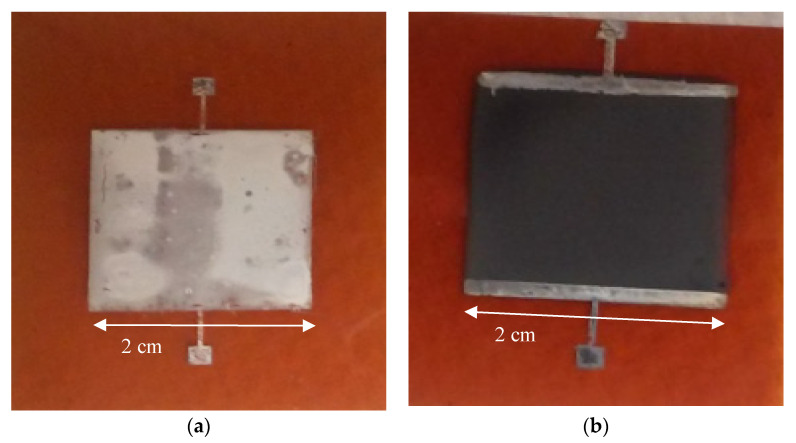
(**a**) Surface of oxidized silver patch. (**b**) Surface of graphene after the oxidation test.

The oxidation test was carried out on a hot plate, in air. Each patch was first heated to 120 °C for one hour. Resistance measurements were taken, and then the process was repeated for 10 °C hourly increases up to 290 °C. Figure 5 shows the data for the oxidation test of resistance changes due to temperature variation for both silver and graphene patches. In Figure 6a,b, obvious oxidization on the surface of silver can be observed. It is observed that the graphene does not change in appearance, and its conductivity was doubled due to thermally activated carriers. This test implies that silver will decay with time, while graphene maintains performance even in harsh environments.

### 3.2. Graphene-Based Phased Array Antennas (PAA) Design and Fabrication

The time delay between adjacent elements of a PAA can be calculated as follows [4]:

(1) $$t\;=\;dsin\theta /ct=\frac{d\sin\theta }{c},$$

where d is the distance between adjacent elements, c is the speed of light, and *θ* is the beam steering angle. The delay line length interval for adjacent antenna elements can be calculated as follows [4]:

*L_interval_ = tc/n*, (2)

where n is the refractive index of the microwave propagation media. 

**Figure 7:**
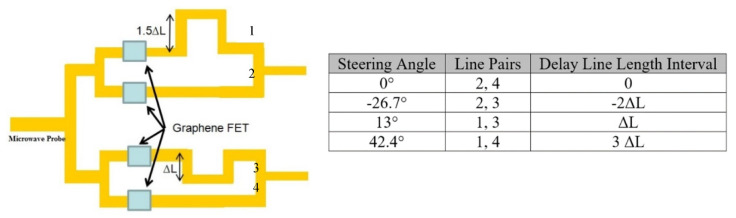
Layout of the 2-bit true-time delay network and designed steering angles.

Using the above equations, we designed a true-time delay network for a PAA radiating at 4 GHz (within S band). Element distance was designed as half wavelength, to avoid grating lobes. Figure 7 shows the layout of the 2-bit true-time delay network and designed azimuth steering angles, which are −26.7°, 0°, 13°, and 42.4°. 

**Figure 8:**
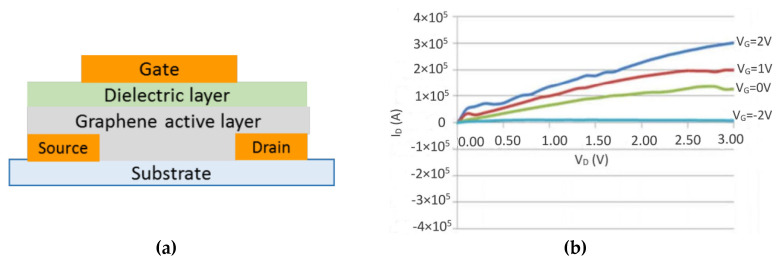
(**a**) Cross-sectional view of the graphene field effect transistor (FET) structure. (**b**) *I_D_*-*V_D_* curves of the printed graphene FET with *V_G_* varied from −2 to 2 v.

The top-gate schematic structure of the proposed graphene FET is shown in Figure 8a. On a flexible DuPont™ Kapton^®^ FPC polyimide film, source and drain electrodes were inkjet printed, using a Fujifilm Dimatix DMP-2800 materials deposition system. Silver nanoparticle ink (Novacentrix Metalon^®^ JS-B40G) (Novacentrix, Austin, TX, USA) was used for electrodes. The printed electrodes were followed by thermal annealing at 130 °C for 30 min. Then, the graphene active layer, prepared as in Section 2, was printed onto the surface of the substrate covering the source and drain electrodes. Forty layers of graphene were printed, to provide uniform coverage of the channel area. The length of the channel was designed to be 100 μm. Since graphene is an intrinsic zero-bandgap material, the on–off ratio with pure graphene active layer is very low [9]. To achieve a higher on–off ratio, the active layer was doped with MoS_2_ monolayer/multilayer dispersion [6]. Ion gel was used as the dielectric layer. The last step was to cover the dielectric layer with the gate electrode. With the alignment mark function, various layers were aligned within 10 µm accuracy. In Figure 8b, the on–off ratio of 38 was achieved when the gate voltage was changed from −2 to 2 v [6]. 

**Figure 9:**
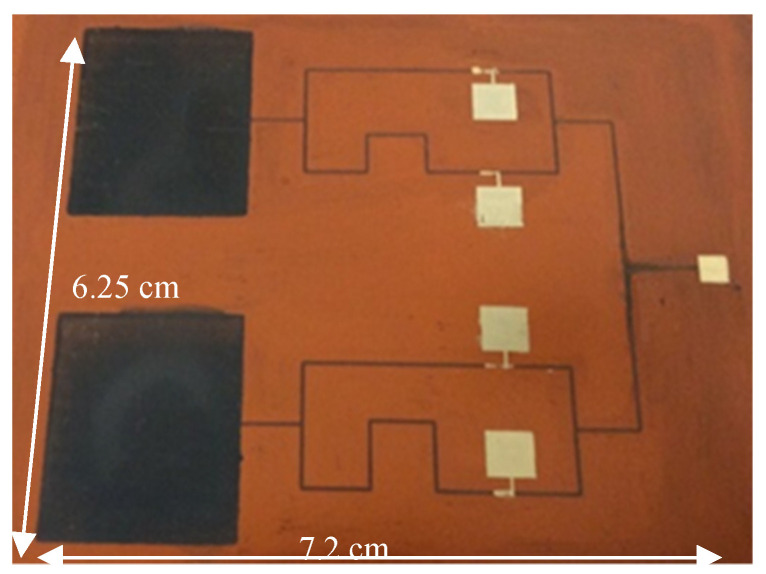
Image of the printed 2-bit 1 × 2 graphene phased array antenna (PAA).

Figure 9 shows the inkjet-printed 2-bit 1 × 2 graphene PAA system with size of 7.2 cm × 6.25 cm. First, all the graphene transmission lines and antenna elements were printed with forty printing passes, to provide good coverage. Then the five graphene transistors were printed layer-by-layer. Finally, the four probes were printed with silver for DC biasing and RF signal coupling. 

### 3.3. Graphene-Based PAA Characterization

**Figure 10:**
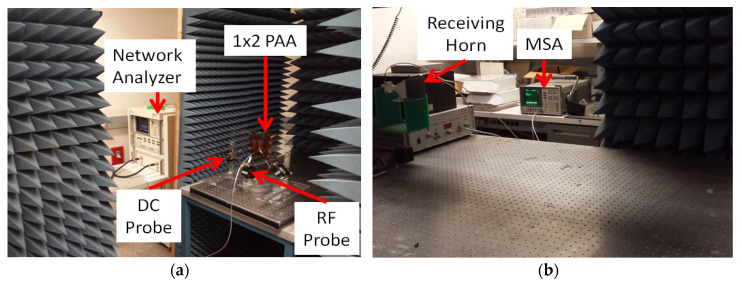
(**a**) Transmitting setup with DC and radio frequency (RF) probing of graphene PAA. (**b**) Receiving measurement, using standard horn and microwave signal analyzer.

Figure 10a,b shows the experimental setup used for far-field measurements of the PAA. The 8510C HP network analyzer provides the S-band signal. Radiation from the antenna array is received by a standard horn antenna with operational frequencies from 3.95 to 5.85 GHz, which can be rotated to measure the far-field pattern of the antenna array. The received signal from the standard horn antenna is read out, using the microwave signal analyzer (MSA) (Keysight Technologies, Santa Rosa, CA, USA). The on/off states of FET switches are controlled, and the far-field pattern at each configuration is recorded. The antenna was tested at 4 GHz. 

**Figure 11:**
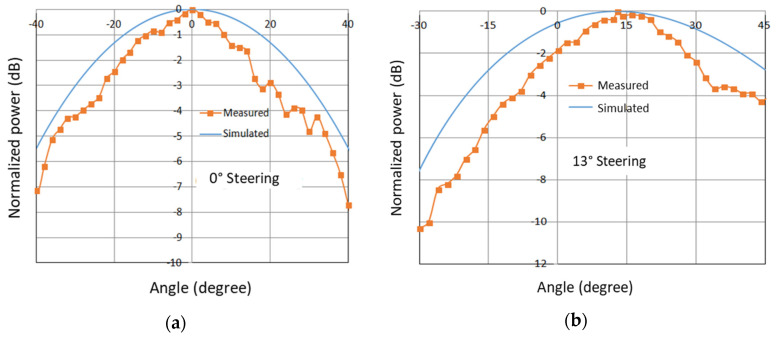
The simulated and measured results of far-field patterns for (**a**) 0° and (**b**) 13° steering.

To demonstrate the azimuth beam steering, Figure 11a,b shows the results for 0° and 13° steering configurations, respectively. The gain of the flexible graphene 2-bit 2-element PAA was measured to be 8.77 dBi, with an efficiency of 47.7%, including the loss of transmission line, FET switch, and coupling loss of RF probes.

## 4. Discussion

In this study, an all-inkjet-printed graphene-based 2-bit 2-element PAA was fabricated on a DuPont™ Kapton^®^ FPC polyimide film, using a Fujifilm Dimatix DMP-2831 inkjet-printing system. Using electronic grade graphene solution, we fabricated an FET with an on–off ratio of 38. The true-time delay lines incorporated with the FET switches were proven to work well through the beam-steering experiments of the PAA. The devices were produced in a room-temperature environment, with thermal curing processes used to anneal the inks which can be substituted with a room-temperature photosintering process. The fully printed graphene PAA technology will greatly help the advancement of conformal communication antennas.

Compared with the mainstream of silver-based printed antennas, graphene antennas take a longer time to be fabricated. Due to very low percentage (less than 1%) of solid loading of graphene ink, it was necessary to continue 40 printing passes in this work, while only three printing passes were needed for our previous silver-based antenna system. To achieve a good conductive graphene film, we performed thermal annealing after every 10 layers, further increasing the fabrication time and process complexity.

## Figures and Tables

**Table 1:** The measured viscosity at different shear rates.

SI No.	Temperature (°C)	Shear Rate (s^−1^)	Regression Coefficient (*R*^2^)	Viscosity (cP)
1	25	150	0.9910	5.57
2	25	372	0.9863	5.62
3	25	750	0.9967	5.51
4	25	1500	0.9890	5.40
5	25	1875	0.9738	5.28

## References

[1] Moussessian A., del Castillo L., Huang J., Sadowy G., Hoffman J., Smith P., Hatake T., Derksen C., Lopez B., Caro E. An active membrane phased array radar. Proceedings of the IEEE MTT-S International Microwave Symposium.

[2] Roy S. (2012). Designing of a Small Wearable Conformal Phased Array Antenna for Wireless Communications. Master’s Thesis.

[3] Salvado R., Loss C., Gonçalves R., Pinho P. (2012). Textile materials for the design of wearable antennas: A survey. Sensors.

[4] Chen M.Y., Pham D., Subbaraman H., Lu X., Chen R.T. (2012). Conformal ink-jet printed-band phased-array antenna incorporating carbon nanotube field-effect transistor based reconfigurable true-time delay lines. IEEE Trans. Microw. Theory Tech..

[5] Subbaraman H., Pham D.T., Xu X., Chen M.Y., Hosseini A., Lu X., Chen R.T. (2013). Inkjet-printed two-dimensional phased-array antenna on a flexible substrate. IEEE Antennas Wirel. Propag. Lett..

[6] Wang Z., Liu Z., Monne M.A., Wang S., Yu Q., Chen M.Y. (2016). Interfacial separation and electrochemical delamination of CVD grown multilayer graphene for recyclable use of Cu powder. RSC Adv..

[7] Monne M.A., Jewel M.U., Wang Z., Chen M.Y. (2018). Graphene Based 3D Printed Single Patch Antenna. Proc. SPIE.

[8] Monne M.A., Enuka E., Wang Z., Chen M.Y. (2017). Inkjet printed graphene-based field-effect transistors on flexible substrate, Low-Dimensional Materials and Devices. Proc. SPIE.

[9] Wang Z., Cook A.F., Yang X., Liu Z., Yu Q., Chen M.Y. (2013). Graphene-based flexible field effect transistor with inkjet printed silver electrodes. Curr. Nanosci..

[10] Pumera M. (2011). Graphene-based nanomaterials for energy storage. Energy Environ. Sci..

[11] Bonaccorso F., Colombo L., Yu G., Stoller M., Tosi M., Ferrari A.C., Ruoff R.S., Pellegrini V. (2015). 2D materials. Graphene, related two-dimensional crystals, and hybrid systems for energy conversion and storage. Science.

[12] Ferrari A.C., Bonaccorso F., Fal’Ko V.I., Novoselov K.S., Roche S., Bøggild P., Borini S., Koppens F.H.L., Palermo V., Pugno N. (2015). Science and technology roadmap for graphene, related two-dimensional crystals, and hybrid systems. J. Nanoscale.

[13] Wang G., Shen X., Yao J., Park J. (2009). Graphene nanosheets for enhanced lithium storage in lithium ionbatteries. Carbon N. Y..

[14] Blake P., Brimicombe P.D., Nair R.R., Booth T.J., Jiang D., Schedin F., Ponomarenko L.A., Morozov S.V., Gleeson H.F., Hill E.H. (2008). Graphene-based liquid crystal device. Nano Lett..

[15] Shao Y., Wang J., Wu H., Liu J., Aksay I.A., Lin Y. (2010). Graphene based electrochemical sensors and biosensors: A review. Int. J. Electroanal..

[16] Enuka E., Monne M.A., Lan X., Gambin V., Koltun R., Tice J., Chen M.Y. (2017). Method of preparing a polar based magnetic ink suitable for inkjet printing and characterization of Ni and Mn ferrite thin film. Adv. Funct. Mater..

[17] Monne M.A., Lan X., Chen M.Y. (2018). Review Article: Material Selection and Fabrication Processes for Flexible Conformal Antennas. Int. J. Antenna Propag..

[18] Soontornpipit P., Furse C.M., Chung Y.C. (2004). Design of implantable microstrip antenna for communication with medical implants. IEEE Trans. Microw. Theory Tech..

[19] Khan A., Nema R. (2012). Analysis of Five Different Dielectric Substrates on Microstrip Patch Antenna. Int. J. Comput. Appl..

[20] Li L.W., Li Y.N., Yeo T.S., Mosig J.R., Martin O.J.F. (2010). A broadband and high-gain metamaterial microstrip antenna. Appl. Phys. Lett..

[21] Elias1 D.C., Nair1 R.R., Mohiuddin T.M.G., Morozov S.V., Blake P., Halsall M.P., Ferrari A.C., Boukhvalov D.W., Katsnelson M.I., Geim A.K. (2009). Control of graphene’s properties by reversible hydrogenation: Evidence for graphene. Science.

[22] Drummond T.G., Hill M.G., Barton J.K. (2003). Electrochemical DNA sensors. Nat. Biotechnol..

[23] Zheng Y., He Z., Gao Y., Liu J. (2013). Direct desktop printed-circuits-on-paper flexible electronics. Sci. Rep..

[24] Monne M.A., Lan X., Zhang C., Chen M.Y. (2018). Inkjet-Printed Flexible MEMS Switches for Phased-Array Antennas. Int. J. Antenna Propag..

[25] Jewel M.U., Monne M.A., Mishra B., Chen M.Y. (2020). Inkjet-printed molybdenum disulfide and nitrogen-doped graphene active layer high On/Off ratio transistors. Molecules.

[26] Jewel M.U., Mahmud M.D.S., Monne M.A., Zakhidov A., Chen M.Y. (2019). Low temperature atomic layer deposition of zirconium oxide for inkjet printed transistor applications. RSC Adv..

[27] Monne M.A., Zaid A., Mia D., Khanal J., Zakhidov A., Chen M.Y. Anti-Reflective Coating for Flexible Devices Using Plasma Enhanced Chemical Vapor Deposition Technique. Proceedings of the 2018 International Conference on Optical MEMS and Nanophotonics (OMN).

[28] Wang Y., Xu H., Zhang J., Li G. (2008). Electrochemical Sensors for Clinic Analysis. Sensors.

[29] Soltman D., Subramanian V. (2008). Inkjet-Printed Line Morphologies and Temperature Control of the Coffee Ring Effect. Am. Chem. Soc. Langmui.

[30] He P., Derby B. (2017). Controlling Coffee Ring Formation during Drying of Inkjet Printed 2D Inks. Adv. Mater. Interfaces.

